# Role of sex and high-fat diet in metabolic and hypothalamic disturbances in the 3xTg-AD mouse model of Alzheimer’s disease

**DOI:** 10.1186/s12974-020-01956-5

**Published:** 2020-09-29

**Authors:** Lisa S. Robison, Olivia J. Gannon, Melissa A. Thomas, Abigail E. Salinero, Charly Abi-Ghanem, Yannick Poitelon, Sophie Belin, Kristen L. Zuloaga

**Affiliations:** Department of Neuroscience & Experimental Therapeutics, Albany Medical College, 47 New Scotland Avenue, MC-136, Albany, NY 12208 USA

**Keywords:** Sex, Alzheimer’s disease, High-fat diet, Obesity, Inflammation, Diabetes, Hypothalamus, Metabolic

## Abstract

**Background:**

Hypothalamic dysfunction occurs early in the clinical course of Alzheimer’s disease (AD), likely contributing to disturbances in feeding behavior and metabolic function that are often observed years prior to the onset of cognitive symptoms. Late-life weight loss and low BMI are associated with increased risk of dementia and faster progression of disease. However, high-fat diet and metabolic disease (e.g., obesity, type 2 diabetes), particularly in mid-life, are associated with increased risk of AD, as well as exacerbated AD pathology and behavioral deficits in animal models. In the current study, we explored possible relationships between hypothalamic function, diet/metabolic status, and AD. Considering the sex bias in AD, with women representing two-thirds of AD patients, we sought to determine whether these relationships vary by sex.

**Methods:**

WT and 3xTg-AD male and female mice were fed a control (10% fat) or high-fat (HF 60% fat) diet from ~ 3–7 months of age, then tested for metabolic and hypothalamic disturbances.

**Results:**

On control diet, male 3xTg-AD mice displayed decreased body weight, reduced fat mass, hypoleptinemia, and mild systemic inflammation, as well as increased expression of gliosis- and inflammation-related genes in the hypothalamus (Iba1, GFAP, TNF-α, IL-1β). In contrast, female 3xTg-AD mice on control diet displayed metabolic disturbances opposite that of 3xTg-AD males (increased body and fat mass, impaired glucose tolerance). HF diet resulted in expected metabolic alterations across groups (increased body and fat mass; glucose intolerance; increased plasma insulin and leptin, decreased ghrelin; nonalcoholic fatty liver disease-related pathology). HF diet resulted in the greatest weight gain, adiposity, and glucose intolerance in 3xTg-AD females, which were associated with markedly increased hypothalamic expression of GFAP and IL-1β, as well as GFAP labeling in several hypothalamic nuclei that regulate energy balance. In contrast, HF diet increased diabetes markers and systemic inflammation preferentially in AD males but did not exacerbate hypothalamic inflammation in this group.

**Conclusions:**

These findings provide further evidence for the roles of hypothalamic and metabolic dysfunction in AD, which in the 3xTg-AD mouse model appears to be dependent on both sex and diet.

## Background

Dementia is estimated to affect nearly 40 million people worldwide, with prevalence expected to triple by the year 2050 [1]. Alzheimer’s disease (AD) is the most common form of dementia, with characteristic brain pathology including amyloid beta (Aβ) plaques and tau tangles believed to start forming decades prior to cognitive decline, in addition to inflammation and neurodegeneration. Although cognitive impairment and associated brain regions (e.g., hippocampus, cortex) have been most widely studied, there are numerous non-cognitive symptoms and associated areas of the brain affected that are commonly observed in AD [2]. Further exploration of these phenomena is necessary to prevent and/or treat non-cognitive complications of AD, which may further aggravate disease progression, contribute to poor quality of life, and may even be life-threatening.

One such group of non-cognitive symptoms seen in AD is metabolic disturbances. Late-life weight loss and low body mass index (BMI) are associated with increased dementia risk, faster disease progression, and increased morbidity and mortality [3–5]. In fact, weight loss is a common clinical feature of AD, reported in up to ~ 40% of cases [6]. Additionally, in both healthy older adults and AD patients, weight loss or low BMI is associated with pathological features of AD, such as increased amyloid burden and CSF biomarkers, as well as cognitive impairment [3, 5, 7–10], suggesting its utility as a biomarker and possible indicator of disease progression. Disturbances in feeding behavior and metabolic function, as well as changes in body weight and composition, are not only seen in AD patients, but also mouse models of the disease. Medications currently approved for AD cannot halt or reverse the progression of the disease, likely because they are begun too late in the disease process. AD patients are usually first diagnosed and begin treatment at the onset of cognitive symptoms, but accumulation of AD pathology in the brain can begin ~ 20 years earlier. Weight loss is sometimes seen in AD patients during the prodromal phase, which can be more than a decade prior to cognitive decline [11–13], highlighting the possibility of using unintentional weight loss or other metabolic markers as early biomarkers of the disease to identify at-risk individuals.

Metabolic disturbances in AD may be linked to changes in hypothalamic function, which plays a role in regulating energy homeostasis, including feeding behavior. Several nuclei in the hypothalamus work together to regulate energy balance, balancing food consumption with energy expenditure. First order neurons that receive peripheral metabolic signals such as insulin, leptin, and ghrelin, in addition to nutrients and metabolites like glucose and fatty acids, lie within the arcuate nucleus (ARC, infundibular nucleus in humans). The two major cell types in the arcuate nucleus express either NPY/AgRP (orexigenic) or POMC/cocaine- and amphetamine-regulated transcript (CART) (anorexigenic). AgRP/NPY and POMC/CART neurons project to other hypothalamic nuclei to regulate food intake and energy expenditure (e.g., locomotor behavior, thermogenesis). This includes the dorsomedial hypothalamus (DMH), lateral hypothalamus area (LHA, major site for hunger), and the ventromedial hypothalamus (VMH, satiety center). Additionally, neurons from the ARC project to the paraventricular nucleus (PVN, a major anorexigenic site), which subsequently regulates food intake, as well as energy expenditure via projections to the nucleus tractus solitarius (NTS) in the brainstem [14]. Overall, the coordinated effort of these hypothalamic nuclei results in signals to other brain regions and the periphery to balance energy intake and expenditure (e.g., control food intake, activity output, thermogenesis) [15].

An accumulation of AD pathology, as well as structural and functional abnormalities, has been noted in the hypothalamus of AD patients, likely contributing to the observed disturbances in energy balance. Both amyloid and tau pathology have been found in several hypothalamic nuclei of AD patients, including those that regulate energy homeostasis [2]. Neuroimaging studies report decreases in hypothalamic volume/gray matter [16–18], perfusion [19, 20], and glucose metabolism [21] in MCI and/or AD patients. Abnormalities in hypothalamic metabolism and cerebral blood flow have also been seen in a mouse model of AD, observed prior to disturbances in the hippocampus and the onset of cognitive decline [22, 23]. Additionally, previous studies suggest that AD pathology may attenuate central responsivity to peripheral metabolic signals. For example, in the Tg2476 mouse model of amyloid pathology, NPY neurons of the arcuate nucleus in the hypothalamus were found to be less responsive to both leptin and ghrelin treatment, an effect that was mimicked by Aβ treatment in slices from WT mice [24]. It appears that the hypothalamus is not only a target of AD pathology, but that metabolic and hypothalamic dysfunction are also key drivers of disease [2]. Hormones involved in the regulation of energy balance have also been shown to specifically affect amyloid processing, tau, and inflammation [25–30]. Additionally, glucose levels, as well as leptin and insulin signaling, not only play a role in maintaining metabolic homeostasis, but they are also vital to maintaining hippocampal function and supporting cognitive processes [31–34]. Therefore, interventions targeting metabolic and hypothalamic dysfunction may also contribute to improved outcomes in AD.

In contrast to late-life weight loss, mid-life obesity and metabolic disease (e.g., type 2 diabetes or prediabetes) are associated with an increased risk for cognitive decline and AD [35, 36]. Metabolic disease and AD are highly comorbid, co-occurring in ~ 80% of patients [37]. Brains of AD patients commonly exhibit defective insulin signaling [38, 39], leading some to argue that AD is actually “Type 3 diabetes” [40]. Consumption of a hypercaloric diet (e.g., high fat, Western) and metabolic disease have been shown to exacerbate AD pathology and related behaviors in rodent models [41–45]. Mechanistically, metabolic disease also causes dysregulation of hormones involved in energy balance, as well as increased inflammation and amyloidosis, which is common to AD [46–48]. The relationship between metabolic disease and AD may be bidirectional, with AD pathology contributing to metabolic alterations [37, 44, 49].

Metabolic disease can cause other pathologies that are linked to AD. One example is nonalcoholic fatty liver disease (NAFLD), a condition in which excess fat accumulates in the liver. It is the most common cause of liver disease in the USA, affecting ~ 30% of adults [50]. Nonalcoholic steatohepatitis (NASH) is a form of NAFLD that also includes inflammation (infiltration of inflammatory immune cells and secretion of pro-inflammatory cytokines) and liver cell damage that can progress to fibrosis (scarring), cirrhosis, and/or liver cancer [51]. NAFLD is associated with increased dementia risk, even in the absence of metabolic disease [52]. NAFLD is associated with altered cerebral blood flow, increased risk and severity of stroke, asymptomatic brain lesions, brain aging, and cognitive impairment [53]. Additionally, the healthy liver plays a role in the peripheral metabolism of amyloid; therefore, in cases of liver disease like NAFLD, impaired peripheral metabolism can reduce the efflux of Aβ from the brain [53]. In fact, animal models of NAFLD show enhanced amyloid and tau pathology, as well as neuroinflammation [54, 55]. However, sex differences in the effects of metabolic disease on NAFLD in AD had not previously been examined.

Despite high rates of comorbidity of metabolic disease in AD patients, little is known about the synergistic effects of AD pathology and high-fat diet on the hypothalamus and peripheral markers of metabolic disease. We explored this relationship in the 3xTg-AD mouse model of AD, which exhibits both amyloid and tau pathology, modeling metabolic disease using a chronic high-fat diet. We also aimed to determine whether any of these effects were sex-dependent, given that women have greater prevalence of AD and obesity particularly in late life [56, 57].

## Methods

### Animals and experimental design

Male and female 3xTg-AD breeder pairs (#34830-JAX) were obtained from Jackson Laboratories (Bar Harbor, Maine) and were used to breed male and female 3xTg-AD mice for this experiment. These 3xTg-AD mice are on a C57BL/6;129X1/SvJ;129S1/Sv genetic background and exhibit three human mutant genes that result in familial AD, including APPSwe, tauP301L, and Psen1^tm1Mpm^ [58]. Intracellular Aβ deposition can be seen as early as 3–4 months of age, in addition to extracellular Aβ deposition, impaired synaptic transmission and LTP by 6 months, and hippocampal deposits of hyperphosphorylated tau at 12–15 months [58, 59]. Male and female B6129SF2/J mice (#101045) were obtained from Jackson Laboratories (Bar Harbor, Maine) to be used as wild-type (WT) controls. At approximately 3 months of age, mice were put on either a HF diet (60% fat, 5.24 kcal/g; D12492, Research Diets, New Brunswick, NJ) or a control diet (CON; 10% fat; D12450B, Research Diets). This age was chosen, as we previously found that starting high-fat diet in young adulthood results in a pre-diabetic phenotype (weight gain and glucose intolerance) that is similar in male and female WT mice [60]. These mice were part of a larger study and received a sham surgery (~ 10 min under isoflurane anesthesia, small incision in the neck, sealed with tissue adhesive), followed by buprenex injections 2× daily for 3 days to alleviate pain. The short sham surgery did not cause weight loss in either sex.

Following 3 months on the diet, mice (*N* = 18–25 per group) underwent a glucose tolerance test (GTT) to assess diabetic status. After 4 months on the diet, mice were deeply anesthetized with anesthesia (~ 4% isoflurane), and blood (cardiac puncture) and tissues were collected. Wet weights were taken of heart, fat pads (visceral and subcutaneous), and reproductive organs (seminal vesicles or uterus). This study was conducted in accordance with the National Institutes of Health guidelines for the care and use of animals in research, and protocols were approved by the Institutional Animal Care and Use Committee at Albany Medical College, Albany, NY, USA.

### Glucose tolerance test

At 3 months post-dietary intervention, mice were fasted overnight, and baseline glucose levels were measured by glucometer (Breeze 2, Bayer, Tarrytown, NY). Each mouse received an i.p. injection of 20% glucose at 10 μL/g of body weight, and blood glucose levels were re-measured at 15, 30, 60, 90, and 120 min.

### Open field test

An open field test was performed after mice were on respective diets for at least 3 months. Mice were placed in a square arena and allowed to explore freely for 10 min, then removed and placed in a “recovery cage” so as not to expose them to naïve cage mates. Distance traveled was assessed as a measure of general activity levels. Thigmotaxis was assessed as a measure of anxiety-like comorbidities. To assess thigmotaxis, the open field arena was divided into a thigmotaxis zone (from the walls of the box to 1/8 of the width of the box) and a central zone (remaining box). Percent of the time spent in the thigmotaxis zone was calculated.

### Plasma diabetes markers and cytokines

Mice had been fasted ~ 5 h prior to euthanasia and blood collection. During euthanasia, blood was collected via cardiac puncture, mixed with 5 μL of EDTA on ice, and spun at 1500×*g* for 10 min at 4 °C to collect plasma. Plasma samples were stored at − 80 °C until assayed. Diabetes-associated markers and cytokine levels in the plasma were assessed using the Bio-Plex Pro Mouse Diabetes 8-Plex Assay (Cat# 171F7001M; Bio-Rad, Carlsbad, California) and the Bio-Plex Pro Mouse Cytokine 23-Plex Assay (Cat# M60009RDPD; Bio-Rad, Carlsbad, California), respectively, according to the manufacturer’s instructions.

### Liver histology

After livers were removed from mice during euthanasia, they were post-fixed for 24 h in 4% formalin, then placed in 30% sucrose solution for 72 h. Livers were frozen in OCT compound and stored at − 80 °C until being cut at 5 μm and stained with either hematoxylin and eosin (H&E) or Sirius Red.

### H&E staining and analysis

Hallmarks of NAFLD, steatosis, ballooning, and inflammation were assessed using hematoxylin and eosin (H&E) staining. Sections were equilibrated to room temperature before being washed in phosphate-buffered saline (PBS) for 5 min. The sections were stained in 0.1% Mayer’s hematoxylin (Sigma MHS-16) for 10 min and 0.5% eosin (Sigma Eosin Y-solution 0.5% alcoholic) for 10 min. They were dipped in 50% EtOH ten times, 70% EtOH ten times, equilibrated in 95% EtOH for 30 s, and in 100% EtOH for 1 min. The sections were then dipped in xylene multiple times, mounted with Cytoseal XYL (Thermo Fisher 8312-4), and cover slipped. Sections were viewed on a Zeiss Primo Vert Inverted Phase Contrast microscope and imaged with an attached Axiocam 105 color camera.

The NAFLD activity score is a semi-quantitative method of analyzing the hallmarks of NAFLD, including steatosis, ballooning, and inflammation. These three attributes characterize the disease, and each category is graded on a scale of 0–3, adapted from a previous study [61]. Steatosis, or microvesicular fat, is defined as faint or patchy white areas within cell cytoplasm. A score of 0 indicates no steatosis while a score of 3 indicates mostly steatosis. Ballooning is defined as larger, circular aggregates of fat between cells (macrovesicular fat). A score of 0 indicates no ballooning while a score of 3 indicates the greatest amount of ballooning. Inflammation is characterized by the clustering of leukocytes resulting in focal inflammation. A score of 0 is no inflammation while a score of 3 is distinctive clusters. See Supplemental Figure 1 in additional materials for representative images for each of these scoring paradigms. Three liver sections per animal were rated to obtain this 3-part score (0–3 for steatosis, ballooning, and inflammation).

#### Sirius red staining and analysis

Sections were equilibrated to room temperature before being incubated in 0.1% Sirius Red (Sigma “Direct Red 80”) for 1 h. The sections were then washed in acidified water twice and dipped in 100% EtOH three times. They were cleared in xylene and mounted with Cytoseal XYL. The sections were viewed on a Zeiss Primo Vert Inverted Phase Contrast microscope and imaged with an attached Axiocam 105 color camera. In ImageJ (NIH), images were converted into 8-bit files and underwent thresholding. Regions of interest were drawn on liver sections, and fibrosis was measured as percent area positive for staining.

### Immunofluorescence

Mice used for immunofluorescence were transcardially perfused with 0.9% saline. Brains were rapidly removed and post-fixed overnight in 4% formalin, then cryoprotected in 30% sucrose for at least 72 h, embedded in OCT, and stored at − 80 °C until cryosectioning. Brains were sectioned in the coronal plane into 6 series of 40-μM thick sections. Sections containing the hypothalamus were washed with PBS with 0.01% sodium azide, permeabilized in 0.3% TPBS with sodium azide for 1 h at room temperature, blocked in 4% donkey serum in 0.3% TPBS with sodium azide for 1 h at room temperature, and incubated at 4 °C overnight with primary antibodies. Primary antibodies for the evaluation of microglia/macrophages included goat anti-Iba1 (1:1000, PA5-18039, ThermoFisher, Lot # TI2638761), while rat anti-glial fibrillary acidic protein (1:2500, AB5804, Millipore, Lot # TA265137) was used for the evaluation of astrocytes. Rabbit anti-NeuN (1:1000, ABN78, Millipore, Lot # 3041797) or DAPI (1:1000; added with secondary antibodies) were used to visualize hypothalamic nuclei and draw regions of interest for analysis (see next section). Fluorescent secondary antibodies (Jackson ImmunoResearch, West Grove, PA), including Rhodamine Red-X Donkey Anti-Rabbit (1:100), Alexa Fluor 647 Donkey Anti-Goat (1:300), and DyLight™ 405 AffiniPure Donkey Anti-Rat (1:300), were diluted in blocking buffer and applied at room temperature for 2 h.

### Immunofluorescence analysis

Images for quantification were taken of the hypothalamus at × 10 using the Axio Observer fluorescent microscope (Carl Zeiss Microscopy, Jena, Germany). All immunohistochemistry analyses were performed in coronal sections across the hypothalamus with 6–7 sections per mouse analyzed (Bregma range − 0.58 to − 2.3 mm). All measurements were performed by an experimenter who was blinded to the identity of the treatment group from which sections came. Iba1 and GFAP images were separately thresholded using the ImageJ (NIH, Bethesda, MD, USA) software. NeuN labeling or DAPI staining were used to visualize hypothalamic nuclei and draw regions of interest. Regions of interest were drawn around the arcuate nucleus (ARC), dorsomedial hypothalamus (DMH), ventromedial hypothalamus (VMH), lateral hypothalamus area (LHA), and paraventricular nucleus (PVN) of every 6th section using ImageJ to quantify the average area covered by cells positive for each of these antibodies.

### Quantitative reverse transcriptase-PCR (RT-qPCR)

During euthanasia, the brains were rapidly removed and hypothalamus microdissected in PBS over dry ice, then stored at − 80 °C until RNA isolation was performed. Total RNA was extracted using the TRIZOL method. All samples containing RNA were treated using the TURBO DNA-free Kit (Invitrogen, Catalog number AM1907) before reverse transcription. cDNA was prepared using 1 μg RNA and the High-Capacity cDNA Reverse Transcription Kit (Applied Biosystems, Catalog number: 4368814). The qPCR reactions were performed using TaqMan Gene Expression Master Mix (Applied Biosystems, Catalog number 4369016) in the presence of Taqman Assays with primer/probes for Iba1 (Mm00479862_g1), GFAP (Mm01253033_m1), IL-1β (Mm00434228_m1), TNF-α (Mm00443258_m1), IL-6 (Mm00446190_m1), AgRP (Mm00475829_g1), NPY (Mm01410146_m1), POMC (Mm00435874_m1), LepR (Mm00440181_m1), MCR4 (Mm00457483_s1), and FNDC5 (Mm01181543_m1) as target genes, and HPRT (Mm01324427_m1), RPL13A (Mm01612986_gH), and RPS17 (Mm01314921_g1) as housekeeping genes. Samples, housekeeping genes, water (negative control), and a reference/positive control sample were run in triplicate. Statistical analyses were performed on ΔCt values, and data was plotted as relative normalized expression compared to male WT control fed mice.

### Statistical analysis

All data are expressed as mean + SEM. Four-way repeated measures ANOVAs (between-subjects measures: AD, sex, diet; within-subject measure: time) were used for analyses of monthly body weight and blood glucose levels over time. Analyses of all other measures were performed using a three-way ANOVA (between-subjects measures: AD, sex, diet). ANOVA’s were followed by post hoc tests (Tukey method) when appropriate. Correlations were run separately for each sex/genotype (WT males, AD males, WT females, AD females) to assess the relationships between metabolic and hypothalamic abnormalities. Statistical significance was set at *p* < 0.05, and statistical analyses were performed using the GraphPad Prism v8, SigmaStat v12, or Statistica v13 software.

## Results

### Sex and diet interact to influence weight gain, adiposity, and glucose intolerance in 3xTg-AD mice

#### Weight gain and adiposity

**Fig. 1:**
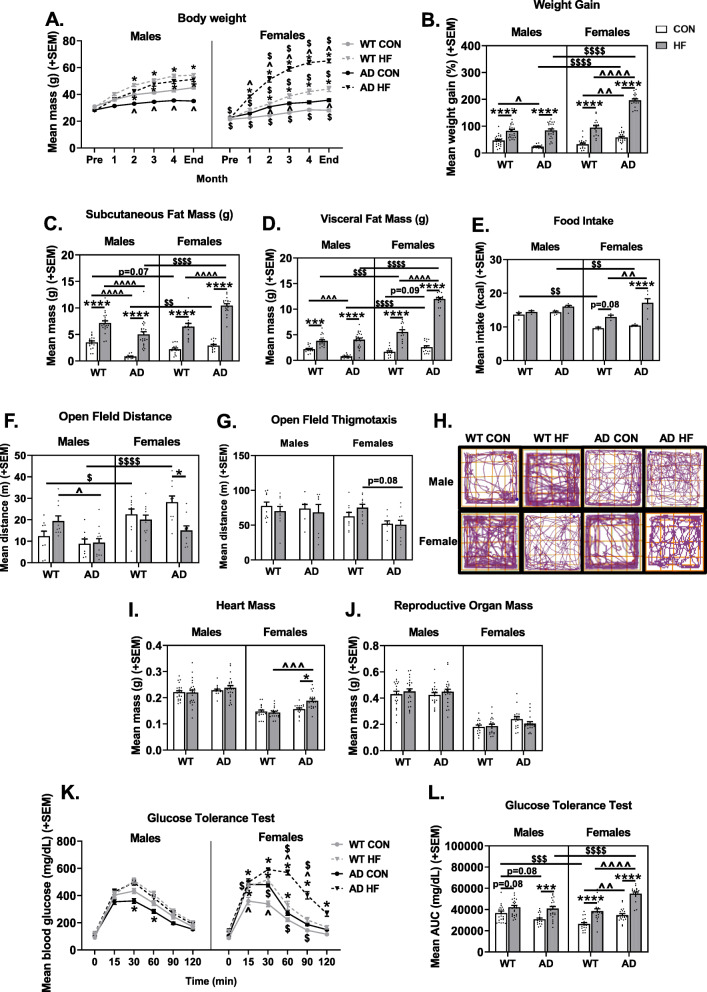
Sex and diet interact to influence weight gain, adiposity, and glucose intolerance in 3xTg-AD mice. **a** Body weight was measured at the beginning of the experiment, prior to the start of respective diet intervention, once per month during the diet intervention, and at the end of the experiment just prior to tissue collection. *N* = 18–25/group. **b** Weight gain was calculated as the percent difference in body weight at the end of the experiment versus initial body weight measured prior to the start of diet intervention. *N* = 18–25/group. **c** Subcutaneous and **d** visceral fat wet weights (in grams) assessed at the end of the experiment. *N* = 14–24/group. **e** Food intake was measured (in grams) to obtain an average measure of daily food intake for each cage of mice within the same treatment group. The mass of food consumed was multiplied by the energy density in each respective diet type to obtain the average daily caloric intake. Each data point represents the mean intake of one cage of mice (3–5 mice per cage). *N* = 3–6 cages/group. **f** Distance traveled (m) in a 10-min open field test. *N* = 8–13/group. **g** Thigmotaxis in a 10-min open field test. *N* = 8-13/group. **h** Representative traces of locomotor behavior in a 10-min open field test. Mean wet weight (in grams) of the heart (**i**) and reproductive organs (seminal vesicles in males, uterine weights in females) (**j**). *N* = 14-25/group. **k**–**l** Glucose tolerance testing (GTT). GTT was performed to assess diabetic status 3 months after the start of diet intervention. **k** Blood glucose levels were measured following overnight fasting (*t* = 0), then mice were injected with glucose challenge and blood glucose levels were measured at 15, 30, 60, 90, and 120 min post-injection. **l** Area under the curve for GTT testing was computed as a measure of total glucose exposure. *N* = 18–25/group. * = diet effect *p* < 0.05; ** = diet effect *p* < 0.01; *** = diet effect *p* < 0.001; **** = diet effect *p* < 0.0001; $ = sex effect *p* < 0.05; $$ = sex effect *p* < 0.01; $$$ = sex effect *p* < 0.001; $$$$ = sex effect *p* < 0.0001; ^ = AD effect *p*<0.05; ^^ = AD effect *p* < 0.01; ^^^ = AD effect *p* < 0.001; ^^^^ = AD effect *p* < 0.0001

To assess sex differences in the metabolic effects of high-fat diet, we measured body weight monthly (Fig. 1a) and calculated weight gain throughout the entire diet intervention (Fig. 1b). Adiposity was assessed by measuring the mass of subcutaneous (Fig. 1c) and visceral (Fig. 1d) fat wet weights upon euthanasia. On control diet, AD males weighed less than WT males (month 2 through the end of the experiment; *p* < 0.05 for all), while the opposite was seen in females, with AD female mice weighing more than WT female mice (month 2, month 3, end weight; *p* < 0.05 for all). On control diet, AD males also had less subcutaneous fat and visceral fat than both WT males (*p* < 0.001) and AD females (*p* ≤ 0.001). In all groups, mice on a HF diet gained significantly higher percentage of weight (*p* < 0.0001 for all), subcutaneous fat (*p* < 0.0001 for all), and visceral fat (*p* < 0.001 for all) compared to their control diet-fed counterparts. Of note, AD HF-fed females gained a higher percentage of weight (*p* < 0.0001 vs. WT HF females and AD HF males), and had more subcutaneous fat (*p* < 0.0001 vs. WT HF females and AD HF males) and visceral fat (*p* < 0.0001 vs. all).

Some of the observed differences in body weight and adiposity could be related to changes in energy intake and expenditure; therefore, food intake (Fig. 1e) and activity levels in an open field test (Fig. 1f) were assessed. WT (*p* = 0.0781) and AD (*p* < 0.0001) females, but not males, on a HF diet consumed more food compared to their control diet-fed counterparts. Additionally, AD HF females ate more than WT HF females (*p* = 0.0035). The open field test revealed that reduced body mass and adiposity observed in AD males compared to WT males was not associated with increased locomotor behavior (*p* > 0.05); in fact, AD HF males were hypoactive compared to WT HF males (*p* = 0.0208). However, increased weight gain and adiposity in AD HF females was indeed associated with reduced activity levels in the open field compared to AD CON females (*p* = 0.0018) (Fig. 1f). Additionally, open field data was analyzed for thigmotaxis (Fig. 1g) as a measure of anxiety-like comorbidities. Females and AD mice showed less thigmotaxis [main effects of sex (*p* < 0.05) and diet (*p* < 0.01)] and there was a trend for less thigmotaxis in AD HF females compared to WT HF females (*p* = 0.08). This could indicate greater exploratory and decreased anxiety-like behavior; however, spatial disorientation and wandering is a non-cognitive symptom seen in Alzheimer’s disease [62, 63]. Diet did not impact perimeter-seeking behavior. Representative traces of open field activity are shown for each group (Fig. 1h).

#### Organ mass

Heart mass was assessed as a surrogate measure of cardiac stress, as it is increased by obesity [53, 54] (Fig. 1i). The HF diet also increased heart mass in AD females (*p* = 0.0235 vs. AD CON female, *p* = 0.0002 vs. HF WT female), but not males.

Reproductive organ weight was assessed as a surrogate measure of sex hormone levels [64–66] (Fig. 1j). Neither AD nor diet significantly affected reproductive organ mass (*p* > 0.10 for all) in either sex, suggesting that it is unlikely that hormone levels were altered by these treatments.

#### Glucose tolerance test

A glucose tolerance test was performed after approximately 3 months on respective diets. Blood glucose levels were assessed immediately prior to and at intervals of up to 2 h after a glucose challenge injection (Fig. 1k). Analysis of area under the curve (AUC) following the glucose challenge was also computed (Fig. 1l). On a control diet, WT females had better glucose tolerance compared to males (*p* = 0.0003). Additionally, on control diet, AD males trended towards having better glucose tolerance compared to WT counterparts (*p* = 0.081). Conversely, AD females had impaired glucose tolerance compared to WT counterparts (*p* = 0.0053). HF diet impaired glucose tolerance in all groups; however, this did not reach significance for WT males (*p* = 0.081; *p* < 0.001 for all others). Of note, HF diet impaired glucose tolerance to the greatest degree in AD females (*p* < 0.0001 vs. WT HF females and AD HF males).

### HF diet and AD are associated with NAFLD pathology

**Fig. 2:**
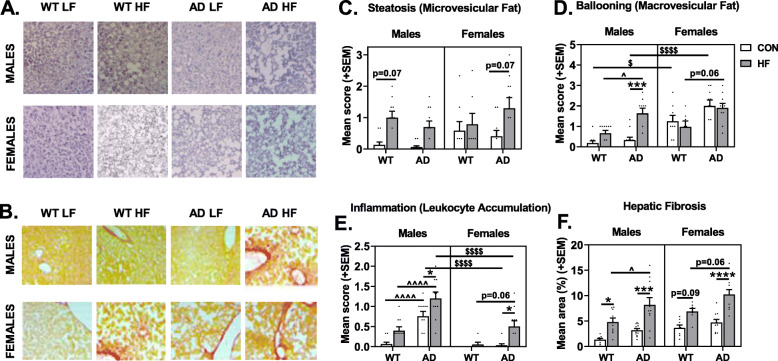
HF diet and AD are associated with nonalcoholic fatty liver disease (NAFLD)-related pathology. Liver sections were stained with **a** hematoxylin and eosin (H&E) or **b** Sirius red to assess NAFLD pathology. H&E-stained sections were assessed for **c** steatosis (microvesicular fat), **d** ballooning (macrovesicular fat), and **e** inflammation (leukocyte accumulation) using a semi-quantitative scoring system (scored 0–3). **f** Sirius red-stained sections were assessed for hepatic fibrosis by measuring the percent area positive for the stain. *N* = 7–12/group for all measures. * = diet effect *p* < 0.05; *** = diet effect *p* < 0.001; **** = diet effect *p* < 0.0001; $$$$ = sex effect *p* < 0.0001; ^ = AD effect *p* < 0.05; ^^^^ = AD effect *p* < 0.0001

We evaluated the presence and severity of NAFLD in these mice not only because of the interrelationship between metabolic disease and NAFLD [67], but also because evidence suggests that NAFLD may contribute to AD [53]. NAFLD was assessed by scoring liver sections stained with hematoxylin and eosin (H&E; Fig. 2a) or Sirius Red (Fig. 2b). H&E-stained sections were scored for steatosis (intracellular microvesicular fat; Fig. 2c), ballooning (extracellular macrovesicular fat; Fig. 2d), and inflammation (leukocyte accumulation; Fig. 2e). Compared to males, females had greater steatosis (trend, *p* = 0.0668) and ballooning (*p* < 0.0001); this sex difference was particularly pronounced in mice on control diet. Conversely, females had lower inflammation scores overall (*p* < 0.0001). As expected, HF diet increased steatosis (*p* = 0.0001) and inflammation (*p* < 0.0001), though HF diet increased ballooning in males only (sex × diet interaction *p* = 0.0011). While there were no differences between WT and AD mice in steatosis, AD mice had greater ballooning (*p* < 0.0001) and inflammation (*p* < 0.0001) compared to WT mice. AD males also appeared to be most susceptible to HF diet-induced NAFLD pathology, including ballooning (*p* = 0.0193 vs. WT HF males) and particularly inflammation (*p* < 0.05 vs. all other groups).

Hepatic fibrosis was assessed by percent area positive for Sirius Red stain (Fig. 2f). As expected, HF diet increased fibrosis (main effect of diet; *p* < 0.0001). Additionally, AD mice had greater fibrosis compared to WT mice (main effect of AD, *p* < 0.0001), and females had greater fibrosis than males (*p* = 0.0009).

### Sex-specific effects of HF diet and AD on plasma levels of diabetes-associated markers

**Fig. 3:**
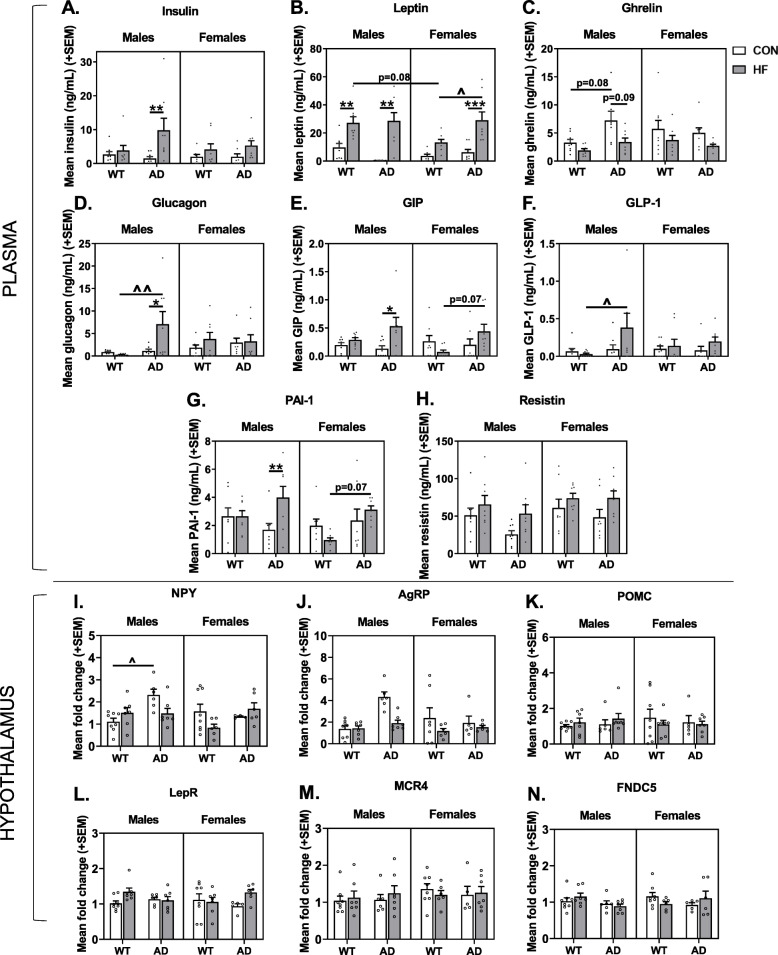
Sex-specific effects of HF diet and AD on plasma levels of diabetes-associated markers and expression of hypothalamic peptides that regulate feeding. **a**–**h** Plasma concentrations of diabetes-related markers. Blood was collected following a 5 h fasting period just prior to euthanasia, and assayed for **a** insulin, **b** leptin, **c** ghrelin, **d** glucagon, **e** gastric inhibitory polypeptide (GIP), **f** glucagon-like-peptide 1 (GLP-1), **g** plasma plasminogen activator inhibitor-1 (PAI-1), and **h** resistin. *N* = 8/group for all plasma markers. **i**–**n** Gene expression levels in homogenate of the whole hypothalamus related to energy balance. Hypothalamus was collected following a 5 h fasting period just prior to euthanasia, and assayed for **i** neuropeptide Y (NPY), **j** agouti-related peptide (AgRP), **k** pro-opiomelanocortin (POMC), **l** leptin receptor (LepR), **m** melanocortin receptor 4 (MCR4), **n** fibronectin type III domain-containing protein 5 (FNDC5). *N* = 5–8/group for all gene expression analyses in the hypothalamus. * = diet effect *p* < 0.05; ** = diet effect *p* < 0.01; ^ = AD effect *p* < 0.05; ^^ = AD effect *p* < 0.01

Plasma was assayed for diabetes-associated markers (Fig. 3a–h). Leptin (Fig. 3b) and ghrelin (Fig. 3c) are hormones that have opposing actions on energy balance via signaling in the hypothalamus. Leptin is primarily secreted by adipocytes to attenuate food intake and promote energy expenditure [68]. Of note, we found that AD males on control diet exhibited severe hypoleptinemia, such that plasma leptin levels were barely detectable. As expected, HF diet resulted in hyperleptinemia (*p* < 0.0001 main effect of diet). This diet effect was significant in WT males (*p* = 0.0141), AD males (*p* < 0.0001), and AD females (*p* = 0.0006), but not WT females. In females on HF diet, AD mice had higher leptin levels than WT mice (*p* = 0.0326). Ghrelin is a fast-acting anorexigenic hormone serving to stimulate feeding behavior [68]. Mice on HF diet had lower ghrelin levels compared to control diet-fed mice (*p* < 0.0001 main effect of diet). Within WT mice, females had higher levels of ghrelin than males (*p* = 0.019), while AD males had higher ghrelin levels than WT males (*p* = 0.015).

Insulin (Fig. 3a) and glucagon (Fig. 3d) are hormones involved in maintaining homeostatic blood glucose levels. While insulin promotes cells absorbing glucose, thus reducing blood glucose levels to prevent hyperglycemia, glucagon signals for the release of stored glucose from the liver, resulting in increased blood glucose levels to prevent hypoglycemia [69]. HF diet resulted in hyperinsulinemia in AD males (*p* = 0.0092). AD HF males also had higher levels of glucagon compared to AD control fed males (*p* = 0.0399) and WT HF males (*p* = 0.0080).

Gastric inhibitory polypeptide (GIP; Fig. 3e) and glucagon-like-peptide 1 (GLP-1; Fig. 3f) are two hormones with opposing actions on postprandial glucagon secretion, such that GIP enhances it and GLP-1 suppresses it. GLP-1 and GIP also act in the hypothalamus to induce satiety and decrease food consumption [70]. AD HF males had higher GLP-1 levels compared to WT HF males (*p* = 0.0363). Levels of circulating GIP also appeared to be highest in AD HF males. Though mice on HF diet had higher levels of GIP compared to control diet-fed mice (*p* < 0.0001 main effect of diet), this was driven by AD HF males which had higher GIP levels compared to their control fed counterparts (*p* = 0.0421), and AD HF females tended to have higher levels than WT HF females (*p* = 0.0743).

Plasma plasminogen activator inhibitor-1 (PAI-1; Fig. 3g) is synthesized by adipose tissue, and increases in PAI-1 can result from inflammatory signals and contribute to a pro-thrombotic state and the development of metabolic disease [71]. HF AD mice had higher levels than WT mice (*p* = 0.002). AD HF males had higher levels than AD control fed males, while AD HF females had higher levels than WT HF females (*p* = 0.0705).

Resistin (Fig. 3h) is a hormone secreted by adipose tissue, as well as immune and epithelial cells, which suppresses insulin’s ability to promote cellular glucose uptake. Mice on HF diet had higher levels of resistin compared to control diet-fed mice (*p* = 0.0050 main effect of diet). Although not significant, this tended to be more pronounced in AD compared to WT mice.

### Hypothalamic expression of orexigenic peptides is increased in AD males on control diet

Several signaling molecules work together in the hypothalamus to regulate energy homeostasis, balancing food consumption with energy expenditure. Relative expression of genes involved in energy balance was assessed in homogenate of the whole hypothalamus (Fig. 3i–n). This included neuropeptide Y (NPY; fast-acting orexigenic peptide; Fig. 3i) and agouti-related peptide (AgRP; delayed, longer-acting orexigenic peptide; Fig. 3j) [72], pro-opiomelanocortin [POMC; an anorexigenic precursor protein and member of the central melanocortin system; cleaved to α-melanocyte stimulating hormone (α-MSH)] [73] (Fig. 3k), leptin receptor (LepR; binds the anorexigenic hormone, leptin; Fig. 3l), melanocortin receptor 4 (MCR4; a G protein-coupled receptor that binds α-MSH; Fig. 3m), and fibronectin type III domain-containing protein 5 (FNDC5; the precursor of irisin, a thermogenic adipomyokine; Fig. 3n) [74].

There was a main effect of AD to increase expression of both NPY (*p* = 0.0058) and AgRP (*p* = 0.0265) compared to WT mice. This effect seems to be primarily driven by control diet-fed AD males expressing higher levels of each gene compared to their WT counterparts, though this reached statistical significance for NPY (*p* = 0.0394; AD × sex × diet interaction *p* = 0.0072) but not for AgRP (*p* = 0.1093). There were no other main effects, interactions, or group differences for hypothalamic gene expression levels of POMC, MCR4, LepR, or FNDC5 (*p* > 0.05 for all).

### Systemic inflammation is increased in AD males only and exacerbated by HF diet

**Fig. 4:**
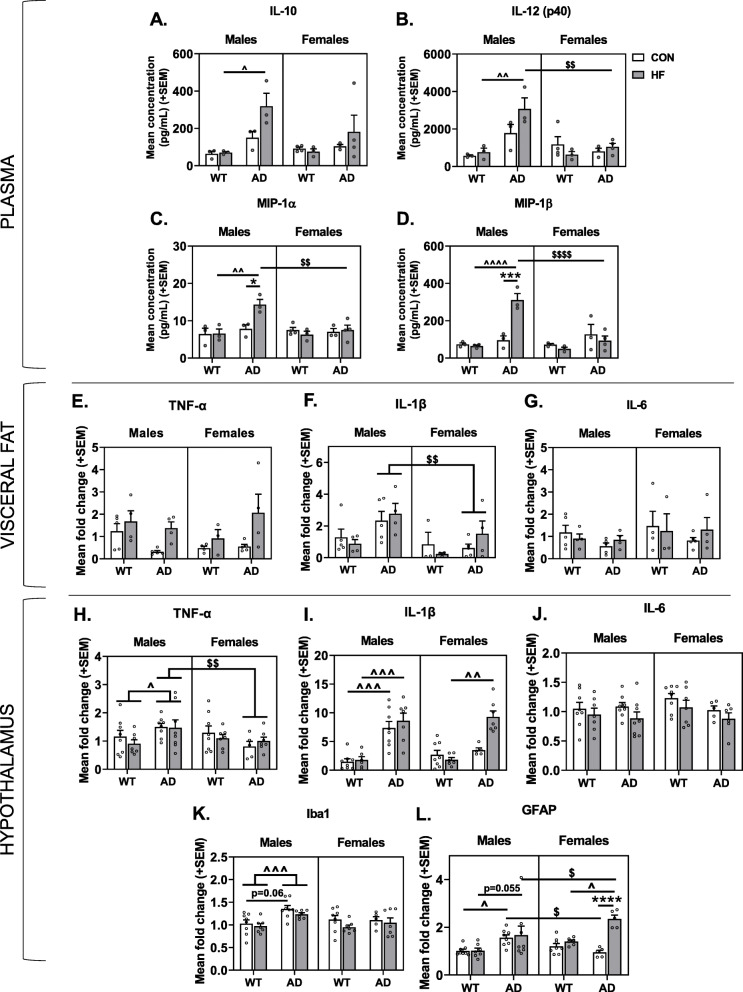
Sex differences in the effects of AD and diet on peripheral inflammation and hypothalamic expression of inflammation-related genes. **a**–**d** Plasma concentrations of cytokines. Blood was collected following a 5 h fasting period just prior to euthanasia and assayed for 23 cytokines, with significant group differences seen for **a** IL-10, **b** IL-12 (p40), **c** MIP-1α, and **d** MIP-1β. *N* = 3–4/group for all plasma markers. **e**–**g** Gene expression levels in visceral fat related to inflammation, including **e** TNF-α, **f** IL-1β, **g** IL-6. **h**–**l** Gene expression levels in homogenate of the whole hypothalamus related to inflammation. Hypothalamus was collected following a 5-h fasting period just prior to euthanasia and assayed for **h** TNF-α, **i** IL-1β, **j** IL-6, **k** Iba1, **l** GFAP. *N* = 5–8/group for all gene expression analyses in the hypothalamus. * = diet effect *p* < 0.05; *** = diet effect *p* < 0.001; **** = diet effect *p* < 0.0001; $ = sex effect *p* < 0.05; $$ = sex effect *p* < 0.01; $$$$ = sex effect *p* < 0.0001; ^ = AD effect *p* < 0.05; ^^ = AD effect *p* < 0.01; ^^^ = AD effect *p* < 0.001; ^^^^ = AD effect *p* < 0.0001

Plasma levels of 23 cytokines were assessed as markers for systemic inflammation. Significant group differences were seen in levels of IL-10, IL-12 (p40), MIP-1α, and MIP-1β (Fig. 4a–d). Cytokines with results of non-significant group differences are shown in Supplemental Table 1. There were increases in IL-10 and IL-12 (p40) in AD mice (*p* < 0.01 for both); this main effect was driven by males and exacerbated by HF diet. Similar trends of increased cytokine levels in HF-fed AD males were seen for MIP-1α and MIP-1β (*p* < 0.05 vs. all other groups).

Visceral fat was also investigated as a potential source of inflammation in the periphery (Fig. 4e–g). Notably, gene expression levels of TNF-α were significantly increased by HF diet in AD mice (*p* = 0.0018) but not WT mice. Additionally, AD males had greater IL-1β expression in visceral fat compared to AD females (*p* = 0.0372). Gene expression levels of IL-6 were not significantly different between groups.

### Sex differences in the effects of AD and diet on hypothalamic expression of inflammation-related genes

Since neuroinflammation is increased in response to HF diet and is believed to contribute to both metabolic disease and neurodegenerative processes in AD [75, 76], we examined relative gene expression for markers of neuroinflammation in hypothalamic tissue (Fig. 4h–l).

AD mice had higher expression of Iba1 (ionized calcium-binding adaptor molecule 1, a microglia marker that increases when microglia are activated [77, 78]) compared to WT mice (main effect of AD *p* = 0.0058). This effect seemed to be driven by males (AD × sex interaction *p* = 0.0351), and AD males had higher expression of Iba1 compared to WT males (*p* < 0.001) and AD females (*p* = 0.014). Additionally, AD males on control diet had a trend toward greater Iba1 expression compared to their WT counterparts (*p* = 0.0603).

AD males on both control (*p* = 0.0272) and HF (*p* = 0.0558) diet had higher GFAP (glial fibrillary acidic protein, indicative of reactive astrocytes [79]) expression compared to their WT counterparts. AD control males also had greater GFAP expression compared to AD control-fed females (*p* = 0.0272). In females, the combination of AD and HF diet resulted in significantly increased GFAP expression compared to all other groups (*p* < 0.05 for all).

Next, we assessed levels of cytokines (TNF-α, IL-1β, and IL-6) that have been implicated in both metabolic disease and AD pathogenesis [80–82]. Mimicking Iba1 results, male AD mice had higher expression of TNF-ɑ compared to WT males (*p* = 0.020) and AD females (*p* = 0.006). Similarly, AD males, regardless of diet, had higher IL-1β expression compared to WT males (*p* < 0.001 for both). AD females only had increased IL-1β expression compared to WT females when on a HF diet (*p* = 0.0020). Lastly, mice on a HF diet had lower expression of IL-6 (*p* = 0.0290 main effect of diet) compared to control-fed mice.

Correlations were run to test the hypothesis that changes in inflammation-related gene expression in the hypothalamus were associated with metabolic disturbances (Table 1). In AD males only, weight gain was inversely associated with IL-6 (*p* < 0.05 for all). In AD females only, weight gain was positively associated with IL-1β and GFAP expression (*p* < 0.05 for all). To explore possible mechanisms of hypothalamic astrogliosis in AD females that are associated with weight gain, we ran correlations between GFAP expression with known astrocyte activators, leptin (plasma) and IL-1β (hypothalamic expression) (Table 1). These correlations were run for all groups, but only in AD females was GFAP expression positively associated with both plasma leptin and hypothalamic expression of IL-1β (*p* < 0.05 for both).

**Table 1:** Sex- and genotype-specific correlations between metabolic outcomes and hypothalamic abnormalities

	WT MALES		AD MALES		WT FEMALES		AD FEMALES	
	*R*	*p*	*R*	*p*	*R*	*p*	*R*	*p*
Weight gain vs. IL-6 (hyp)	− 0.049	0.863	− **0.587**	0.017*	− 0.201	0.473	− 0.395	0.230
Weight gain vs. IL-1β (hyp)	0.201	0.491	0.280	0.312	− 0.401	0.155	**0.806**	0.0012**
Weight gain vs. GFAP (hyp)	− 0.231	0.408	− 0.164	0.544	0.217	0.456	**0.891**	< 0.0001****
Leptin (plasma) vs. GFAP (hyp)	− 0.239	0.454	− 0.333	0.267	0.182	0.593	**0.756**	0.030*
IL-1β (hyp) vs. GFAP (hyp)	0.004	0.988	0.043	0.878	0.432	0.140	**0.935**	< 0.0001****
Weight gain vs. Iba1 (PVN)	0.031	0.933	0.704	0.051	− 0.069	0.870	**0.775**	0.009**
Weight gain vs. GFAP (ARC)	− 0.022	0.956	− 0.005	0.992	0.490	0.151	**0.780**	0.008**
Weight gain vs. GFAP (DMH)	**0.759**	0.018*	**0.755**	0.030*	**0.854**	0.002**	**0.932**	< 0.0001****
Weight gain vs. GFAP (VMH)	0.237	0.510	0.389	0.341	0.501	0.140	**0.834**	0.003**
Weight gain vs. GFAP (PVN)	− 0.169	0.663	0.357	0.432	− 0.074	0.839	**0.857**	0.003**

Correlations were run for each sex/genotype (WT males, AD males, WT females, AD females), combining mice across control and high fat diets, to explore relationships between metabolic outcomes and hypothalamic abnormalities. *p < 0.05, **p < 0.01, ***p < 0.001, ****p < 0.0001

### Sex differences in hypothalamic gliosis in response to AD and HF diet

Since gene expression for Iba1 and GFAP was increased in the homogenate of the whole hypothalamus, immunofluorescence was performed to localize specific nuclei that experience gliosis in response to AD and HF diet. Although amyloid deposition was not observed in any hypothalamic region at this age point (data not shown), gliosis was observed.

#### Iba1 labeling

**Fig. 5:**
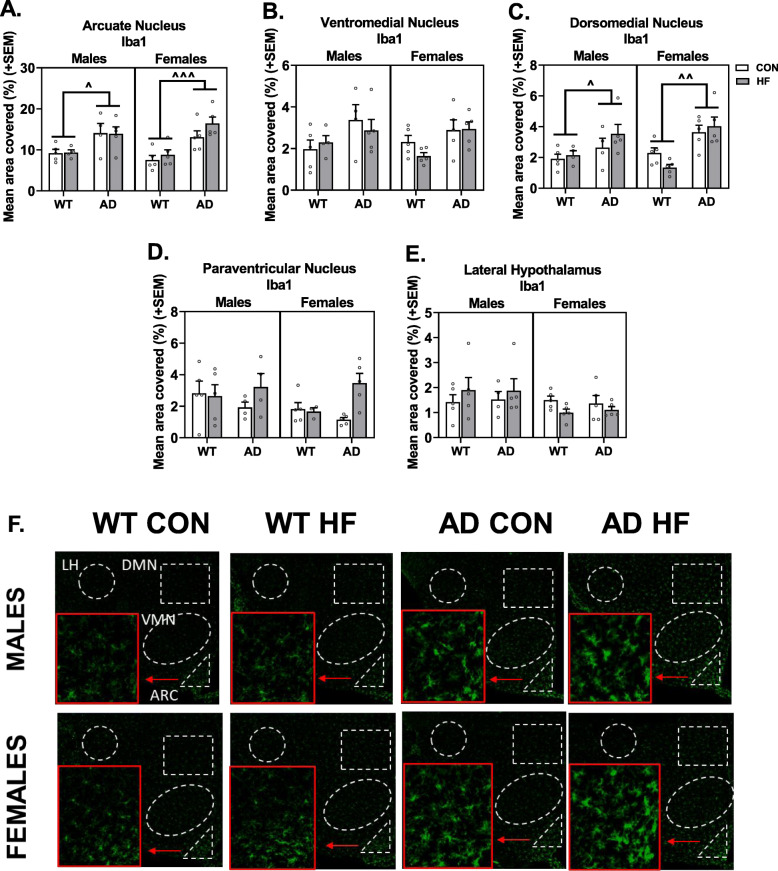
Hypothalamic microgliosis in AD mice occurs in a nucleus-specific manner but is unaffected by sex or HF diet. Labeling of tissue sections including the hypothalamus was performed for Iba1. Quantification was performed for percent area positive for each label in the arcuate nucleus (**a**), ventromedial nucleus (**b**), dorsomedial nucleus (**c**), paraventricular nucleus (**d**), and lateral hypothalamus area (**e**). Representative images for labeling with Iba1 are also shown (**f**). *N* = 3–5/group. ^ = AD effect *p* < 0.05; ^^ = AD effect *p* < 0.01; ^^^ = AD effect *p* < 0.001

Immunolabeling was performed for Iba1 to assess microgliosis in nuclei of the hypothalamus that regulate energy balance (Fig. 5a–f). Iba1 immunoreactivity was increased in AD mice in the ARC (*p* < 0.0001), DMH (*p* < 0.0001), and VMH (*p* = 0.0040). Additionally, HF diet increased Iba1 labeling in the PVN of AD mice, but not WT mice (AD × diet interaction *p* = 0.0271). No group differences were seen in Iba1 labeling in the LHA.

Correlations were run to test the hypothesis that hypothalamic microgliosis was associated with metabolic disturbances (Table 1). Correlations were seen in the PVN only, and this finding was restricted to AD mice. In both male and female AD mice, Iba1 labeling in the PVN was positively associated with weight gain, though this reached statistical significance in females only.

#### GFAP labeling

**Fig. 6:**
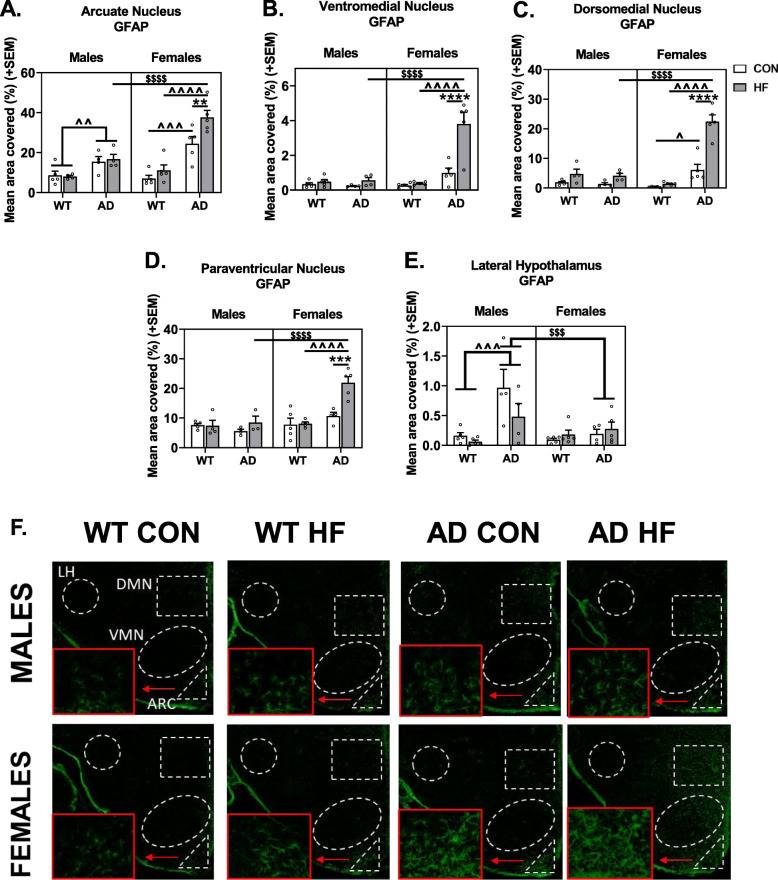
Sex differences in hypothalamic gliosis in response to AD and HF diet. Labeling of tissue sections including the hypothalamus was performed for GFAP. Quantification was performed for percent area positive for each label in the arcuate nucleus (**a**), ventromedial nucleus (**b**), dorsomedial nucleus (**c**), paraventricular nucleus (**d**), and lateral hypothalamus area (**e**). Representative images for labeling with GFAP are also shown (**f**). *N* = 3–5/group. *** = diet effect *p* < 0.001; **** = diet effect *p* < 0.0001; $$$ = sex effect *p* < 0.001; $$$$ = sex effect *p* < 0.0001; ^^ = AD effect *p* < 0.01; ^^^ = AD effect *p* < 0.001; ^^^^ = AD effect *p* < 0.0001

Immunolabeling was performed for GFAP to localize astrogliosis in hypothalamic nuclei that regulate energy balance (Fig. 6a–f). In the arcuate nucleus, HF diet resulted in an increase in reactive astrocytes in females but not males (*p* = 0.002). AD mice had greater area covered by GFAP-positive label compared to WT mice, in both males (*p* = 0.009) and females (*p* < 0.001). The HF diet-induced increase in GFAP was greater in AD females compared to males (*p* < 0.001), with AD HF females having the greatest GFAP coverage of all groups.

Trends of GFAP labeling were similar within the DMH, VMH, and PVN. In these regions, there was a main effect of HF diet mice having greater coverage than control diet (*p* < 0.01 for all). Additionally, AD females had greater GFAP labeling than both WT females and AD males (AD × sex interaction *p* < 0.0001). The most striking effect was seen in AD HF females, having ~ 3–4× or more GFAP coverage than all other groups in these three regions (*p* < 0.0001 for all).

There was a clearly different trend in the LHA, with a significant AD × sex interaction (*p* = 0.0089) showing that AD males had greater GFAP coverage compared to WT males and AD females (*p* ≤ 0.001 for both).

Correlations were run to test the hypothesis that hypothalamic astrogliosis was associated with metabolic disturbances (Table 1). Positive associations were seen between GFAP labeling in the DMH and weight gain in all groups (WT male, AD male, WT female, AD female). However, only in AD females was weight gain positively associated with GFAP labeling in the ARC, VMH, and PVN.

## Discussion

Metabolic abnormalities are associated with increased risk of AD, which may be both a result of and contribute to AD pathology, hypothalamic dysfunction, and behavioral deficits [2]. In the current study, we investigated the interaction between AD and HF diet to influence peripheral and hypothalamic measures of metabolism and inflammation in male and female 3xTg-AD mice. Exploring sex differences in these relationships was of interest given the sex bias of AD (females more greatly affected than males) [48] and to determine whether the potential utility of any findings as novel biomarkers or targets for treatment may be sex-specific. Here, we report that AD males and females exhibit abnormalities in body/fat mass and glucose intolerance in opposite directions (increased in females, decreased in males). AD males exhibit widespread increases in peripheral and hypothalamic inflammation, with the former exacerbated by HF diet for some measures. HF diet in AD females results in severe astrogliosis and increased IL-1β expression in the hypothalamus. These findings may represent two metabolic phenotypes (weight loss and obesity/weight gain) seen in AD patients, with different physiological and/or neurobiological mechanisms.

We observed drastic sex differences in metabolic status of 3xTg-AD mice on a control diet relative to WT mice, with males exhibiting weight loss but females exhibiting weight gain. Along with weight loss, adult male 3xTg-AD mice also exhibit decreased fat mass and better glucose tolerance compared to their WT counterparts. This is in line with clinical findings that report unintentional weight loss in up to ~ 40% of cases [6]. Conversely, AD females exhibited metabolic disturbances opposite to males, such that AD females displayed increased body and fat mass, in addition to impaired glucose tolerance. This is in line with AD patients having heightened risk for metabolic disease such as type 2 diabetes [37]. Additionally, in mice, intracerebroventricular injections of Aβ oligomers lead to peripheral glucose intolerance [49]. Our finding of decreased weight in males is in line with a prior study in another AD mouse model (Tg2576 mice) [24]. However, our finding of increased weight in females is in contrast to that study, which also found deficits in body mass and fat in Tg2576 females [24]. Altered body mass (decreased in males and increased in females) in our 3xTg-AD mice was not observed at the start of the experiment (3–4 months of age), suggesting metabolic disturbances progress with disease status. Although clinically important weight *loss* is more common in AD patients compared to normal older controls, this same study also found that significant weight gain (> 5%) was more likely to occur in AD patients [83], suggesting that two metabolic phenotypes may exist in AD patients, which may be represented by our male and female mice.

The weight-loss metabolic phenotype we observed in control-fed males was not explained by differences in activity levels or food intake but was accompanied by several endocrine changes, such as low plasma leptin and increased plasma ghrelin, that may explain this weight loss. Hypoleptinemia has been demonstrated in AD patients [84] and mouse models [24], observed prior to AD plaques [24], and with severity becoming more pronounced through the course of the disease. Here, we report nearly non-existent levels of circulating leptin in control-fed AD males. Hypoleptinemia may be due not only to the presence of less adipose tissue, but also attenuated leptin secretion by adipocytes, as seen in mouse models of AD [85]. Impaired leptin signaling is not only believed to be a result of AD pathology, but it may also further promote AD pathology and cognitive deficits. In AD mouse models, low leptin is associated with impaired cognitive function and increased Aβ burden [86–88]. Clinical studies have shown that hypoleptinemia is associated with decreased hippocampal gray matter [89] and cognitive decline in older adults [90]. Leptin has been explicitly shown to play a role in maintaining proper structure and function of the hippocampus [91] and is protective against Aβ and tau pathology [27]. In line with low circulating leptin, our control-fed male AD mice also exhibit increased plasma ghrelin and increased hypothalamic expression of NPY and AgRP. Higher circulating ghrelin levels are associated with poorer cognitive performance [92], and levels of the activated form of ghrelin are elevated in MCI patients [93]. However, there is little to no evidence to support a causative effect; in contrast, ghrelin may be neuroprotective [94–96]. Taken together, these findings support a growing body of evidence that normalizing metabolic dysfunction and perhaps specifically leptin signaling could prove to be a novel target for treatment of AD [31].

Our AD female mice, but not male mice, showed a phenotype of enhanced susceptibility to metabolic impairment in response to HF diet. Few studies have assessed sex differences in the effects of HF diet in AD mice, despite the sex bias in AD [56], high rates of comorbidity of metabolic disease and AD in humans [37], and that HF diet (particularly saturated fat) and metabolic dysfunction are believed to contribute to AD risk and progression [35, 36]. Here, we report that WT and AD males had similar weight and glucose intolerance on HF diet; however, AD females were considerably more susceptible to increases in body and fat mass, as well as glucose intolerance, in response to HF diet compared to all other groups. AD HF-fed females displayed ~ 2–3× as much visceral fat compared to all other groups on the same diet. Visceral fat is thought to be the “bad” fat, contributing to inflammation, disease risk (increased risk of diabetes, cardiovascular disease, stroke), and is linked to cognitive deficits and dementia risk above and beyond obesity [97]. In line with increased adiposity, AD HF females also exhibited hyperleptinemia compared to other female groups, which may have contributed to glucose intolerance. AD HF females also exhibited increased heart mass, likely to support blood flow throughout a body with increased mass; this is in line with previous findings suggesting that HF diet/obesity are associated with increased cardiac stress [98, 99]. While differences in energy intake or output (activity levels) could not explain findings in AD females on control diet, AD females on HF diet consumed a greater number of calories and were hypoactive, which contribute to an energy surplus state. Our 3xTg-AD model may be a model of “accelerated aging,” in which case our findings are in line with our previous studies reporting that female mice exhibit increased weight gain and glucose intolerance on HF diet compared to males in middle age (~ 15 months) [60, 100]. Interestingly, we also found that HF diet caused a wider array of cognitive deficits in middle-aged WT females compared to males [100] and that HF impairs adult hippocampal neurogenesis in females but not males [101]. Ongoing studies in our lab will determine if these sex differences in cognitive effects extend to AD and multi-etiology dementia. Overall, our current findings provide additional evidence for increased susceptibility to diet-induced metabolic dysregulation in the presence of AD pathology, which has been reported in another AD mouse model [102].

We examined NAFLD pathology because metabolic disease is known to promote NAFLD [51] and because increasing evidence suggests that NAFLD may, in turn, contribute to AD [53]. AD males and females accumulated similar amounts of microvesicular and macrovesicular fat in the liver in response to high-fat diet, though AD mice overall had greater macrovesicular fat and hepatic fibrosis. Surprisingly, AD females showed elevated macrovesicular fat even on a control diet. It is possible that this increased macrovesicular fat in AD females on control diet could have contributed to their glucose intolerance, as liver fat content shows greater association with glucose intolerance than even visceral fat [103]. Of note, AD males had greater leukocyte accumulation, a sign of potential inflammation in the liver, compared to other groups; this was exacerbated by HF diet. Increased leukocyte accumulation in the liver in response to HF diet was also seen in AD females, albeit to a lesser degree. These findings were somewhat surprising, given results on weight gain, adiposity, and glucose tolerance in these groups. However, more severe NAFLD pathology in AD males on HF diet was in line with findings that several other plasma markers associated with diabetes were disproportionately increased in this group, despite adiposity that was less than or equal to other HF-fed groups. Despite AD HF females exhibiting increased weight gain, adiposity, and glucose intolerance compared to all other groups, many other metabolic markers in circulation or expression in the hypothalamus were not seemingly disproportionately altered. This is in line with a previous study in 3xTg-AD mice showing males but not females exhibit hyperinsulinemia in response to HF diet; however, these males also displayed increased ectopic fat despite similar weight gain and hyperglycemia, in contrast to current findings [43]. NAFLD is characterized by a pro-inflammatory state, which may at least in part be linked to the increased systemic inflammation (plasma IL-10, IL-12, MIP-1α, and MIP-1β) seen in AD males and exacerbated by HF diet in this group, mimicking trends of increased leukocyte accumulation in the liver. WT mice of both sexes lacked a severe peripheral or central inflammatory response to HF diet that was seen in AD mice. Though generally associated with chronic low-grade inflammation, our study joins others in reporting minimal inflammatory effects of chronic HF diet in WT mice [104].

Both AD and metabolic disease are linked to chronic systemic inflammation, which can lead to tissue damage and impaired function. Specifically, hypothalamic inflammation in obesity is thought to contribute to impaired action of leptin, insulin, and other hormones [105]. In the current study, increased inflammation in the periphery of male AD mice (liver, visceral fat, and plasma) mimics trends of increased hypothalamic expression of Iba1, GFAP, and pro-inflammatory cytokines (TNF-a and IL-1β). Previous studies have also reported increased hypothalamic expression of pro-inflammatory markers in 3xTg-AD male mice at even 12 weeks of age [106]. Additionally, free fatty acids from the consumption of a HF diet can contribute to hypothalamic inflammation [107]. Inflammation and impaired liver function are hypothesized to contribute to BBB breakdown, which would allow for the infiltration of peripheral immune cells [53]. Of note, the purposeful leakiness of the blood brain barrier in the mediobasal hypothalamus, which normally functions to allow the sensing of peripheral signals, also leaves it particularly vulnerable to this source of inflammation. Additionally, microglia are targets of circulating cytokines, including liver-derived metabolic inflammation, promoting further neuroinflammation through the release of proinflammatory cytokines [108]. In fact, NAFLD can result in significant increases in brain levels of TNF-α and IL-6, as well as neurodegeneration [109]. Immunohistochemical analyses also revealed that AD males exhibited microgliosis and astrogliosis in several hypothalamic nuclei involved in energy balance, a phenomenon that was largely unaffected further by HF diet. In contrast to AD males, AD females did not exhibit significant increases in plasma markers of inflammation, even on HF diet. AD HF females did exhibit moderately elevated liver inflammation, as well as increased hypothalamic expression of only IL-1β; however, this was to an equal or lesser degree compared to male counterparts. Our findings may be reflective of theories that high-grade inflammation is associated with unintentional weight loss (as in control AD males, counteracted by HF diet) while low-grade inflammation is associated with obesity/metabolic disease [105, 110].

The most striking difference we observed in the hypothalamus of AD females, particularly on a HF diet, was a marked increase in GFAP expression. In addition to contributing to inflammation under obesogenic conditions, glial cells play a multifaceted role in energy balance, potentially in a sex-specific manner [111]. Although GFAP labeling in the DMH was correlated with weight gain in all groups, GFAP labeling in the ARC, VMH, and PVN, and GFAP expression in the whole hypothalamus was positively correlated with metabolic outcomes such as weight gain in AD females only. These findings suggest that the metabolic deficits in AD females and enhanced susceptibility to HF diet may be at least in part related to astrogliosis in these hypothalamic nuclei. Astrocytes can become reactive in response to leptin and IL-1β; both of which were increased in AD females. This proposed mechanism is supported by our findings that both plasma leptin and hypothalamic IL-1β expression were positively correlated with hypothalamic expression of GFAP, and IL-1β expression was associated with metabolic outcomes in AD females only. Astrocytes are capable of ensheathing neurons in the arcuate nucleus, rendering them less responsive to metabolic signaling [112]. In AD HF females, this could contribute to their inability to sense signals trying to maintain energy and glucose homeostasis. Although previous studies suggest that amyloid is also capable of attenuating hypothalamic neuron reactivity to leptin and ghrelin [24], evidence from both mouse models and AD patients suggest that hypothalamic impairment and metabolic abnormalities precede amyloid and tau pathology [2, 113]. This is supported by the current study, in which we do not observe hypothalamic amyloid deposition but do observe hypothalamic inflammation and impairment of function. Therefore, other mechanisms must be playing a role. Astrogliosis and the release of pro-inflammatory cytokines may be contributing to the two metabolic phenotypes reflected in male and female 3xTg-AD mice in the current study; however, further investigation is necessary to confirm and elucidate these possible mechanisms.

## Conclusions

In summary, we found marked sex differences in metabolic outcomes of AD mice both on control and HF diet, which may be representative of two metabolic phenotypes associated with Alzheimer’s disease. We found that on control diet, AD males and females exhibit metabolic changes in opposite directions, with males representing an energy deficit state unattributable to energy intake/output, and females an energy surplus. Our results confirm findings that both weight loss/low body mass index and weight gain/glucose intolerance may be possible biomarkers for AD. Hypothalamic inflammation in control-fed AD mice was worse in males than females, in line with some increases in markers of peripheral inflammation. In males, we see a somewhat “normal” metabolic response to HF diet and no additive effect on neuroinflammation, though there is a clear exacerbation of systemic inflammation and some peripheral markers of metabolic disease. In AD females, we see a dramatic increase in susceptibility to some metabolic effects of HF diet (weight gain, adiposity, glucose intolerance) associated with increased caloric intake and decreased energy expenditure, as well as hyperleptinemia, that is associated with severe astrogliosis in the hypothalamus. Further exploration is necessary to elucidate these potential mechanisms and determine possible new targets for treatment.

## Supplementary information

**Additional file 1:.** Supplemental Table 1. Means and SEMs of plasma cytokines lacking significant group differences. Supplemental Figure 1. Liver scoring. Healthy tissue received a score of 0 in all categories. For ballooning, steatosis, and inflammation, a score of 1 indicates mild pathology, a score of 2 indicates moderate pathology, and a score of 3 indicates severe pathology. Ballooning is large, circular aggregates of fat that displace cells (examples are indicated by arrows in ballooning score panel). It is also referred to as Macrovesicular fat. Steatosis is characterized by white patchy areas within the cytoplasm of the cell (examples are indicated by arrows in the steatosis score panel). This is sometimes referred to as microvesicular fat. Inflammation is defined as lymphocytic infiltrate within the sinusoids and clustered by veins or arteries (examples are indicated by arrows in the inflammation score panel).

## Data Availability

The datasets used and/or analyzed during the current study are available from the corresponding author on reasonable request.

## Abbreviations

α-MSH: Alpha-melanocyte-stimulating hormone
Aβ: Amyloid-beta
AD: Alzheimer’s disease
AgRP: Agouti-related peptide
ANOVA: Analysis of variance
ARC: Arcuate nucleus
AUC: Area under the curve
BMI: Body mass index
CART: Cocaine- and amphetamine-regulated transcript
CON: Control (diet)
CSF: Cerebrospinal fluid
DMH: Dorsomedial hypothalamus
EDTA: Ethylenediaminetetraacetic acid
FNDC5: Fibronectin type III domain-containing protein 5
GFAP: Glial fibrillary acidic protein
GIP: Gastric inhibitory polypeptide
GLP-1: Glucagon-like peptide-1
GTT: Glucose tolerance test
H&E: Hematoxylin and eosin
HF: High fat (diet)
Iba1: Ionized calcium-binding adaptor molecule 1
IL-1β: Interleukin-1 beta
IL-6: Interleukin-6
IL-10: Interleukin-10
IL-12 (p40): Interleukin-12 (p40)
LepR: Leptin receptor
LHA: Lateral hypothalamus area
MCR4: Melanocortin receptor 4
MIP: Macrophage inflammatory protein
NAFLD: Nonalcoholic fatty liver disease
NPY: Neuropeptide Y
PBS: Phosphate saline buffer
POMC: Pro-opiomelanocortin
PVN: Paraventricular nucleus
SEM: Standard error of the mean
TNF-α: Tumor necrosis factor alpha
VMH: Ventromedial hypothalamus
WT: Wild-type
