# Supplementary material for: Role of sex and high-fat diet in metabolic and hypothalamic disturbances in the 3xTg-AD mouse model of Alzheimer’s disease
Source: J Neuroinflammation. 2020 Sep 29;17:285. doi: 10.1186/s12974-020-01956-5 (PMC7526387; doi:10.1186/s12974-020-01956-5)
Supplement: Supplementary file 1 — Additional file 1:. Supplemental Table 1. Means and SEMs of plasma cytokines lacking significant group differences. Supplemental Figure 1. Liver scoring. Healthy tissue received a score of 0 in all categories. For ballooning, steatosis, and inflammation, a score of 1 indicates mild pathology, a score of 2 indicates moderate pathology, and a score of 3 indicates severe pathology. Ballooning is large, circular aggregates of fat that displace cells (examples are indicated by arrows in ballooning score panel). It is also referred to as Macrovesicular fat. Steatosis is characterized by white patchy areas within the cytoplasm of the cell (examples are indicated by arrows in the steatosis score panel). This is sometimes referred to as microvesicular fat. Inflammation is defined as lymphocytic infiltrate within the sinusoids and clustered by veins or arteries (examples are indicated by arrows in the inflammation score panel). [file 12974_2020_1956_MOESM1_ESM.docx]

**ADDITIONAL MATERIALS**

**Supplemental Table 1. Means and SEMs of plasma cytokines lacking significant group differences**

|  | WT M CON | | WT M HF | | AD M CON | | AD M HF | | WT F CON | | WT F HF | | AD F CON | | AD F HF | |
| --- | --- | --- | --- | --- | --- | --- | --- | --- | --- | --- | --- | --- | --- | --- | --- | --- |
| Cytokine | Mean | SEM | Mean | SEM | Mean | SEM | Mean | SEM | Mean | SEM | Mean | SEM | Mean | SEM | Mean | SEM |
| IL-1α | 14.15 | 1.06 | 13.09 | 4.77 | 14.90 | 1.86 | 11.07 | 3.07 | 10.11 | 1.81 | 9.14 | 1.75 | 16.27 | 3.13 | 12.76 | 3.98 |
| IL-1β | 13.06 | 0.30 | 13.34 | 3.92 | 25.51 | 12.63 | 28.24 | 13.35 | 22.08 | 3.05 | 14.61 | 3.16 | 25.98 | 12.52 | 18.94 | 9.67 |
| IL-2 | 6.62 | 0.75 | 11.91 | 4.25 | 13.50 | 6.92 | 7.24 | 0.34 | 15.05 | 6.53 | 6.88 | 1.05 | 10.16 | 4.05 | 5.60 | 0.81 |
| IL-3 | 3.02 | 0.29 | 2.71 | 0.69 | 3.32 | 1.45 | 2.45 | 0.53 | 3.41 | 0.17 | 2.50 | 0.45 | 3.62 | 1.37 | 2.55 | 0.45 |
| IL-4 | 2.35 | 0.19 | 3.54 | 1.28 | 2.73 | 0.95 | 4.16 | 2.38 | 2.89 | 0.40 | 2.40 | 0.64 | 3.18 | 1.06 | 3.70 | 1.81 |
| IL-5 | 12.21 | 3.43 | 13.07 | 6.04 | 9.75 | 3.66 | 14.15 | 6.89 | 16.99 | 5.76 | 16.08 | 5.11 | 7.95 | 1.68 | 10.87 | 3.18 |
| IL-6 | 4.40 | 0.09 | 6.04 | 1.99 | 6.72 | 1.68 | 7.17 | 1.23 | 5.17 | 0.49 | 3.60 | 0.30 | 4.15 | 1.50 | 4.56 | 0.74 |
| IL-9 | 46.23 | 17.08 | 37.43 | 8.80 | 33.41 | 12.39 | 97.93 | 72.04 | 75.23 | 40.74 | 23.65 | 3.96 | 123.47 | 64.01 | 62.86 | 33.73 |
| IL-12 (p70) | 137.16 | 0.66 | 107.72 | 37.47 | 119.96 | 58.32 | 137.99 | 17.15 | 154.74 | 21.43 | 96.13 | 23.86 | 132.21 | 12.19 | 131.87 | 41.23 |
| IL-13 | 84.95 | 4.52 | 102.18 | 28.44 | 221.33 | 119.30 | 150.32 | 30.48 | 356.30 | 183.34 | 110.25 | 19.32 | 231.15 | 109.95 | 117.28 | 28.71 |
| IL-17 | 104.78 | 21.40 | 130.26 | 70.02 | 60.36 | 39.37 | 69.21 | 34.64 | 92.52 | 19.96 | 40.52 | 18.21 | 59.80 | 13.13 | 88.32 | 16.05 |
| Eotaxin | 1224.00 | 124.76 | 955.36 | 237.83 | 292.59 | 98.51 | 416.57 | 143.98 | 1104.08 | 52.49 | 1017.97 | 205.90 | 1225.34 | 319.34 | 845.62 | 240.94 |
| G-CSF | 130.33 | 13.59 | 224.18 | 130.09 | 215.13 | 77.25 | 133.56 | 12.59 | 135.99 | 16.56 | 128.79 | 34.85 | 111.44 | 19.49 | 192.54 | 39.32 |
| GM-CSF | 42.28 | 1.87 | 35.23 | 8.34 | 44.76 | 15.82 | 30.58 | 3.12 | 60.32 | 8.55 | 40.71 | 7.29 | 41.08 | 4.91 | 35.00 | 6.50 |
| IFN-γ | 47.72 | 9.77 | 36.60 | 7.78 | 27.95 | 11.87 | 157.18 | 132.48 | 87.88 | 44.85 | 27.21 | 6.54 | 107.79 | 59.26 | 62.67 | 30.28 |
| KC | 117.77 | 47.73 | 65.49 | 12.59 | 118.21 | 45.74 | 144.24 | 35.67 | 111.28 | 31.13 | 65.96 | 15.89 | 76.91 | 14.70 | 107.76 | 24.46 |
| MCP-1 | 223.95 | 6.72 | 291.75 | 154.58 | 309.96 | 157.48 | 288.75 | 37.71 | 231.80 | 31.60 | 196.30 | 25.82 | 210.55 | 45.59 | 165.34 | 36.89 |
| RANTES | 63.09 | 22.53 | 61.75 | 12.37 | 160.46 | 118.08 | 69.47 | 18.02 | 91.12 | 10.73 | 66.41 | 14.57 | 88.81 | 20.91 | 48.90 | 15.13 |
| TNF-α | 113.04 | 13.81 | 70.86 | 18.11 | 184.99 | 124.10 | 154.34 | 39.60 | 140.62 | 21.50 | 81.58 | 16.45 | 202.78 | 111.03 | 121.11 | 34.06 |

Abbreviations: IL, Interleukin; G-CSF, Granulocyte colony-stimulating factor; GM-CSF, Granulocyte-macrophage colony-stimulating factor; IFN-γ, Interferon-gamma; KC, Keratinocyte chemoattractant; MCP-1, Monocyte chemoattractant protein-1; RANTES, Regulated upon activation, normal T cell expressed and secreted; TNF-α, Tumor necrosis factor alpha

**Supplemental Figure 1. Liver scoring.** Healthy tissue received a score of 0 in all categories. For ballooning, steatosis, and inflammation, a score of 1 indicates mild pathology, a score of 2 indicates moderate pathology, and a score of 3 indicates severe pathology. Ballooning is large, circular aggregates of fat that displace cells (examples are indicated by arrows in ballooning score panel). It is also referred to as Macrovesicular fat. Steatosis is characterized by white patchy areas within the cytoplasm of the cell (examples are indicated by arrows in the steatosis score panel). This is sometimes referred to as microvesicular fat. Inflammation is defined as lymphocytic infiltrate within the sinusoids and clustered by veins or arteries (examples are indicated by arrows in the inflammation score panel).
